# Anti-HIV-1 activity, protease inhibition and safety profile of extracts prepared from *Rhus parviflora*

**DOI:** 10.1186/1472-6882-13-158

**Published:** 2013-07-04

**Authors:** Manoj Modi, Nutan, Boskey Pancholi, Shweta Kulshrestha, Ajay Kumar Singh Rawat, Swadesh Malhotra, Satish Kumar Gupta

**Affiliations:** 1 Reproductive Cell Biology Laboratory, National Institute of Immunology, Aruna Asaf Ali Marg, New Delhi 110 067, India; 2 National Botanical Research Institute, Rana Pratap Marg, Lucknow 226 001, Uttar Pradesh, India

**Keywords:** *Rhus parviflora*, Cytotoxicity, Lactobacilli, Vaginal keratinocytes, Anti-HIV-1 activity, HIV-1 protease, Pro-inflammatory cytokines

## Abstract

**Background:**

In the present study, extracts prepared from the leaves of *Rhus parviflora* Roxb. (Anacardiaceae) were evaluated for their anti-HIV activity, which have been traditionally used for the treatment of neurological disorders such as anxiety, insomnia and epilepsy.

**Methods:**

Aqueous and 50% ethanolic extracts prepared from leaves of the plant were tested for their cytotoxicity and anti-HIV property using reporter gene based assays as well as human peripheral blood lymphocytes (PBLs). Further these extracts were evaluated for their ability to inhibit HIV-1 reverse transcriptase (RT) and protease activity. Safety profile of the extracts was determined on viability of *Lactobacillus* sp., secretion of pro-inflammatory cytokines by vaginal keratinocytes and transepithelial resistance.

**Results:**

Both aqueous (IC_50_ = 15 μg/ml) and 50% ethanolic (IC_50_ = 26 μg/ml) extracts prepared from leaves of *R. parviflora* showed anti-HIV activity in TZM-bl cells wherein the virus was treated with the extracts prior to infection. Further, both the extracts also inhibited virus load in HIV infected CEM-GFP cells and human PBLs. The anti-HIV activity is mediated through inhibition of HIV-1 protease activity. Both the extracts did not disturb the integrity of monolayer formed by intestinal epithelial Caco-2 cells. The extracts when tested up to 100 μg/ml did not significantly reduce the viability of *L. plantarum*, *L. fermentum, L. rhamnosus* and *L. casei*. The extracts (100 μg/ml) did not reveal any cytotoxic effect on vaginal keratinocytes (Vk2/E6E7). Levels of pro-inflammatory cytokines secreted by Vk2/E6E7 cells treated with both the plant extracts were within the non-inflammatory range.

**Conclusions:**

The studies reported herein showed *in vitro* anti-HIV activity and preliminary safety profile of the extracts prepared from the leaves of *R. parviflora*.

## Background

Acquired Immunodeficiency Syndrome (AIDS), an immunological disorder characterized by abnormalities of immunoregulation and opportunistic infections, caused by the human immunodeficiency virus (HIV) is one of the major public health problems [1]. Treatment of HIV infected patients with currently available highly active anti-retroviral (HAART) drugs though successful in reducing the burden of the disease but is associated with various side effects, including emergence of drug resistant HIV strains [2-6]. Hence, it is imperative to discover novel anti-HIV agents from natural sources that may have lesser side effects. Various studies have shown anti-HIV properties of the extracts prepared from variety of plants [7-9]. The plant extracts or purified phytochemicals may exhibit anti-HIV activity by inhibiting virus entry/fusion, HIV-1 reverse transcriptase (RT), protease or its integrase activity [10-13]. Further, to prevent sexual transmission of HIV, microbicides with anti-HIV properties have been proposed that can be applied topically before sexual act [14].

*Rhus parviflora* Roxb. (Anacardiaceae) is known as ‘Tintidika’ in Sanskrit language, widely distributed in Nepal, Northern India, Bhutan and Sri Lanka at the altitudinal range of 700–1100 m [15]. It is recorded in Ayurvedic pharmacopoeia as having therapeutic uses for Vāta vikāra, the complications related to neurological disorders including anxiety, insomnia, epilepsy, and rheumatoid arthritis [16]. In Nepal, fruits of *R. parviflora* are also used for human consumption and decoction of fruit or stem bark used to cure dysentery [17,18]. Bark extract is applied externally on wounds and small twigs are used for cleaning teeth [19]. In some tribal areas, infusions of leaves were given in cholera [20]. Phytochemicals like gallic acid, some flavones viz., rutin, myricetin, quercetin, myricitrin, quercitrin, kampferol and some glycosides (isorhamnetin-3-α-L-arabinoside) have been isolated from the plant [16,21].

The current study was undertaken to evaluate anti-HIV property of the aqueous and 50% ethanolic extracts prepared from leaves of *R. parviflora* using *in vitro* assays. Further, pre-clinical safety profile of these extracts with respect to viability of *Lactobacillus* sp., epithelial cell monolayer integrity and secretion of pro-inflammatory cytokines by vaginal keratinocytes has been studied.

## Methods

### Collection of plant material

Fresh leaves (1 kg) of the wild *R. parviflora* plant were collected in May 2008 from Khairna, Nainital, India (Accession Number-NBRH16) and specimen has been submitted to Herbarium of National Botanical Research Institute (NBRI), Lucknow, India. The plant material was collected and identified by Dr. A. K. S. Rawat, who is a taxonomist/botanist, Pharmacognosy Department, NBRI, Lucknow. The leaves were air and shade dried, grinded and strained through a mesh (size 30, mesh opening 0.5 mm).

### Preparation of 50% ethanolic and aqueous extracts

To prepare 50% ethanolic extract, *R. parviflora* leaves powder (100 gm) was charged in a percolator, treated with ethanol: water (500 ml, 1:1 v/v) and left overnight at 25-30°C. The percolate (300 ml) was drained and the marc extracted thrice by cold percolation, each time with 500 ml of ethanol: water (1:1 v/v) and the combined percolate (1200 ml) was evaporated at 40-45°C under vacuum to concentrate the extract up to 80 ml. The concentrated 50% ethanolic extract was lyophilized at −20 to −40°C to afford 8-10% dried extract.

To prepare aqueous extract, *R. parviflora* leaves powder (100 gm) was treated with 500 ml of MilliQ water at 65-75°C for 6–8 h. The hot water extract was filtered through Whatman filter paper number 1. The marc was extracted thrice, each time with 500 ml of water at 60-75°C. The combined filtrate (1200 ml) was distilled at 45-50°C under vacuum to afford concentrated aqueous extract up to 70 ml. The extract was subsequently, lyophilized at −20 to −40°C to afford 9-11% dried extract. Both aqueous and 50% ethanolic extracts were characterized by High Performance Liquid Chromatography (HPLC), wherein 20 μl of the respective extract (1 mg/ml) was resolved by C18 column (Cap cell Pak C18, Phenomenex, CA, USA) using an isocratic acetonitrile and water supplemented with 10 mM formic acid (35:65; v/v), at a flow rate of 0.4 ml/min. The elution profile was monitored at 280 nm.

### Cell maintenance and HIV

Anti-HIV assays were performed using TZM-bl [recombinant HeLa cell line expressing high levels of CD4, HIV-1 co-receptors CCR5 & CXCR4 with β-galactosidase and luciferase reporter genes under HIV-1 long terminal repeat (LTR) promoter] and CEM-GFP [a CD4^+^ T-lymphocytic reporter cell line expressing green fluorescent protein (GFP) under HIV-1 LTR promoter] reporter cells. TZM-bl cells were maintained in Dulbecco's modified Eagle’s medium (DMEM; Sigma-Aldrich Inc., St. Louis, MO, USA) supplemented with 10% fetal bovine serum (FBS; Biological Industries, Kibbutz Beit Haemek, Israel) and antibiotic-antimycotic cocktail [Penicillin (100 units/ml), Streptomycin (100 μg/ml) and Amphotericin B (250 ng/ml); Pen-Strep-Ampho sol, Biological Industries] whereas, CEM-GFP cells in RPMI-1640 medium (Sigma-Aldrich Inc.) supplemented with 10% FBS, G418 (500 μg/ml; Gibco, Grand Island, NY, USA) and antibiotic-antimycotic cocktail as used for TZM-bl cells [22]. Vk2/E6E7 cells (immortalized cell line derived from the normal human vaginal mucosa), a generous gift from Dr. Raina Fichorova (Brigham and Women’s Hospital, Boston, MA, USA), were cultured in Keratinocyte serum-free medium (ker-sfm) supplemented with bovine pituitary extract and epidermal growth factor (Gibco-Invitrogen, Carlsbad, CA, USA). Caco-2 cells (American Type Culture Collection, Manassas, VA, USA) were cultured in RPMI medium, supplemented with 10% FBS and previously used antibiotic-antimycotic cocktail. Saquinavir (Catalog Number 4658) was obtained from AIDS Research and Reference Reagent Program, Division of AIDS, National Institute of Allergy and Infectious Diseases, USA.

HIV-1_NL4.3_ was prepared by transfection of HEK-293T cells with pNL4.3 plasmid (Catalog number 114; AIDS Research and Reference Reagent Program, Division of AIDS, National Institute of Allergy and Infectious Diseases, USA) using CaPO_4_ method as described previously [23].

### Cytotoxicity assay using MTT

The cytotoxicity of plant extracts on various cell lines was assessed by MTT [3-(4,5-dimethylthiazol-2-yl)-2,5-diphenyltetrazolium bromide; Sigma-Aldrich Inc.] assay [24]. In brief, cells were seeded (6 × 10^3^ adherent cells/well; 5 × 10^4^ suspension cells/well) in 96-well cell culture plates (Greiner Bio-One, GmbH, Frickenhausen, Germany) and grown overnight at 37°C in humidified atmosphere of 5% CO_2_. After 24 h, cells were treated with varying concentrations of the extracts ranging from 10–400 μg/ml**,** for the duration as used to determine the anti-HIV activity [TZM-bl cells - 48 h; CEM-GFP cells - 8 days with a change of medium on 5^th^ day and human peripheral blood lymphocytes (PBLs) for 5 days]. Negative control included cells treated with solvent/medium. After incubation, cell viability was assessed by adding 20 μl MTT (5 mg/ml in 50 mM PBS) per well and incubated at 37°C for 3 h followed by addition of MTT solvent (100 μl/well; 20% SDS and 50% dimethyl formamide in 50 mM PBS) [24]. The absorbance (OD) was read at 570 nm with reference filter at 690 nm. Experiments were performed in duplicates and percent viability was calculated by dividing the OD obtained in treatment group by OD of untreated cell control multiplied by hundred.

### Anti-HIV activity using TZM-bl cells

In TZM-bl cells-based assay, HIV-1_NL4.3_ viral strain at a multiplicity of infection (MOI) of 0.05 was treated with varying concentrations (2–50 μg/ml) of extracts for 1 h at 37°C. Subsequently, HIV-1 pretreated with plant extracts was added to TZM-bl cells (4 × 10^4^/well; seeded on the previous day in 24-well plate) and incubated for 4 h. Subsequently, cells were washed with cold 50 mM PBS, fresh culture medium with extracts added and further incubated for 48 h in humidified atmosphere of 5% CO_2_ at 37°C. Azidothymidine (AZT; Sigma-Aldrich Inc.) was used as positive reference control. After incubation, cells were washed twice with PBS and lysed with 1X lysis buffer (Promega Corporation, Madison, WI, USA). Supernatant was collected and luciferase activity was measured using white optiplate in the Fluorimeter (BMG Labtech GmbH, Offenberg, Germany). The results were expressed as percentage inhibition, calculated by taking the luminescence in experimental group divided by the luminescence in infected cells in absence of test extracts/AZT multiplied by hundred. Percent inhibition was calculated by subtracting the above value from hundred.

### Inhibition of HIV infection using CEM-GFP cells-based assay

CEM-GFP cells (5 × 10^6^) were infected with HIV-1_NL4.3_ virus at an MOI of 0.05 in presence of polybrene (2 μg/ml) for 4 h at 37°C with intermittent mixing as described previously [25]. Post-infection, the cells were washed twice with serum free RPMI-1640 medium and were seeded (2.0 × 10^5^/well) in a 24-well plate. Plant extracts at varying concentrations (1–50 μg/ml) were added to their respective wells. AZT was used as a positive control whereas solvents used to prepare extracts were used as negative controls. On 5^th^ day, 0.4 ml of cell suspension was removed from each well, 1 ml fresh medium along with the extracts was added to the wells and plate was further incubated. On 8^th^ day, 100 μl supernatant was collected for p24 analysis. For GFP estimation, cells were lysed with 150 μl of 1X Promega cell culture lysis buffer and lysate was centrifuged at 9000 × g for 10 min at 4°C. The supernatant (100 μl/well) was transferred to black optiplate and the absorbance was measured at an excitation wavelength of 485 nm and emission at 520 nm using Fluorimeter (FLUostar Optima, BMG Labtech, Germany). The results were expressed as percentage inhibition, calculated by taking the GFP fluorescence in experimental group divided by GFP fluorescence in infected cells in the absence of test extract/AZT multiplied by hundred. Percent inhibition was obtained by subtracting the above value from hundred.

### Anti-HIV assay using human peripheral blood lymphocytes (PBLs)

All experiments using human blood cells were carried out under informed consent of the blood donors and following the clearance from the Institutional Bio-safety and Human Ethical Committee. Blood (5 ml) was taken from healthy HIV sero-negative donors and peripheral blood lymphocytes (PBLs) were isolated using Ficoll density gradient method. Cells (2 × 10^6^ cells/ml) were stimulated for 3 days with 3 μg/ml phytohemagglutinin (PHA-P; Sigma-Aldrich Inc.) and after stimulation washed twice to remove PHA-P. Stimulated cells were infected by HIV-1_NL4.3_ at an MOI of 0.05, in presence of IL-2 (10 U/ml) for 4 h as described for CEM-GFP cells. Infected cells were washed twice with plain medium to remove the unbound virus and seeded in 96-well plate (5 × 10^4^ cells/well), in 200 μl of RPMI medium supplemented with 10% FBS and 10 U/ml recombinant human IL-2. The extracts (20 μl/well) at varying concentrations, diluted in culture medium, were added in duplicate. Cells were cultured at 37°C, 5% CO_2_ and culture supernatant was collected on day 5 for p24 analysis.

The viral load in the supernatant (diluted 1:10 or 1:20) of CEM-GFP cells as well as human PBLs treated with plant extracts was measured using ELISA kits (SAIC-Frederick Inc., NCI-Frederick, USA; XpressBio, Life Science Products, MD, USA) for p24 estimation, following the instructions of the manufacturer. The non-specific inhibition of p24 binding to its antibody in ELISA in the presence of plant extracts was taken into account while calculating p24 concentration in the culture supernatants. Results were expressed as percent inhibition in virus load calculated by dividing the p24 concentration in the presence of plant extracts/AZT by p24 value observed in negative control, multiplied by hundred and the obtained value was subtracted from hundred.

### HIV reverse transcriptase (RT) and protease assay

The inhibitory activity of plant extracts on HIV-1 RT was determined by commercial kit (Roche Applied Sciences, Mannheim, Germany) as per the manual’s instructions. In addition, effect of plant extracts on HIV-1 protease activity was also determined using kit (Anaspec, CA, USA) as per the manufacturer’s instructions.

### Estimation for pro-inflammatory cytokines secreted by human cervico-vaginal keratinocytes

To study the toxic and inflammatory responses of the plant extracts, a human cervico-vaginal keratinocyte cell line (Vk2/E6E7) was used [26]. Cells (6.0 × 10^3^ cells/well) were seeded in 96-well culture plate and incubated in humidified atmosphere of 5% CO_2_ at 37°C for 24 h. After incubation, cells were treated with plant extracts (100 μg/ml) for 24 h and culture supernatant was collected for various cytokines quantiation using BDTM Cytometric Bead Array kit (BD FACSCanto Flow Cytometer; BD Biosciences Pharmigen, San Diego, CA, USA). The kit allows simultaneous quantification of interleukin (IL)-1β, IL-6, IL-8 and tumor necrosis factor (TNF). The cytokine bead assay was performed according to the manufacturer's specifications and data analysis was done using BD FACSDiva software. In addition, Vk2/E6E7 cells viability after 24 h treatment with test extracts was also determined by MTT assay as described above.

### Transepithelial resistance (TER) measurement

Effect of the plant extracts on epithelial cells integrity (transepithelial resistance; TER) was measured using voltmeter. Caco-2 cells (5 × 10^5^ cells/well) were grown in transwells and culture medium was dispensed in the basolateral compartment of each well. The cells were allowed to grow for 36–48 h in 5% CO_2_ at 37°C. Resistance was measured using Millicell–ERS voltmeter (EMD Millipore Corporation, Billerica, MA, USA) each day until resistance reached plateau. After formation of monolayer, extracts (50 μg/ml) was added in the culture medium and cells further incubated in humidified atmosphere of 5% CO_2_ at 37°C. Resistance was measured at intervals of 0.5, 1, 2, 4, 8 and 24 h (cells were incubated in 5% CO_2_ at 37°C in between each reading).

### Effect of plant extracts on the viability of lactobacilli

Various lactobacilli strains such as *Lactobacillus casie* (MTCC 1423), *L. fermentum* (MTCC 903), *L. plantarum* (MTCC 4462) and *L. rhamnosus* (MTCC 1408) were obtained from Institute of Microbial Technology, Chandigarh, India and cultured in MRS broth (HiMedia, Mumbai, India). The cytotoxicity of plant extracts on lactobacilli was assessed by MTT assay as described previously [27,28]. In brief, bacterial density was adjusted to an OD of 0.06 at a wavelength of 670 nm i.e. approximately 10^8^ CFU/ml. Extracts were administered at concentrations ranging from 3.125-100 μg/ml into 96-well plates along with 30 μl of bacterial suspension. Final volume was made up to 100 μl using MRS broth. Negative control included cells treated with solvent/medium and Saquinavir, a known HIV-1 protease inhibitor was used as reference control. After incubation for 24 h at 37°C, 10 μl of MTT (5 mg/ml in 50 mM PBS; Sigma-Aldrich Inc.) was added to each well containing microbial inoculums and plant extracts. Plates were incubated for 3 h at 37°C, followed by centrifugation at 2500 g for 10 min. Supernatants were aspirated and 100 μl of acid-isopropanol (5 ml of 1 N HCl in 95 ml of isopropanol) was added to each well. Optical density was measured using micro plate spectrophotometer (EL_X_ 800MS; BioTek Instrument Inc., Vermont, USA) at 540 nm using reference filter at 690 nm. Percent viability was calculated by dividing the absorbance of treated cells to untreated cells multiplied by hundred.

### Statistical analysis

Analyses of concentration-response data were performed by the use of nonlinear curve-fitting program Prism (Graph Pad Software Inc., CA, USA) to determine CC_50_ and IC_50_ values. The results were average of 2–3 independent experiments. The statistical significance of the values obtained in different assays in presence of varying concentrations of the plant extract with respect to untreated group was calculated using one way ANOVA. A p-value of <0.05 was considered to be statistically significant.

## Results and discussion

With the aim to discover new plants as a source for prevention of HIV infection, aqueous and 50% ethanolic extracts prepared from leaves of *R. parviflora* were evaluated for anti-HIV activity using reporter gene-based cells assays and human PBLs. The safety of the extracts with respect to the effect on epithelial cell integrity, adverse effect on the viability of lactobacilli as well as production of pro-inflammatory cytokines by vaginal keratinocytes was assessed. Analyses of the aqueous and 50% ethanolic extracts prepared from the leaves of *R. parviflora* by HPLC revealed these to be a complex mixture of phytochemicals (Additional file 1: Figure S1A, S1B). It is likely that the aqueous extract will have preponderance of polar compounds whereas 50% ethanolic extract will have higher concentration of non-polar compounds.

### Aqueous and 50% ethanolic extracts from leaves of *R. parviflora* inhibit HIV-1 infection

**Figure 1:**
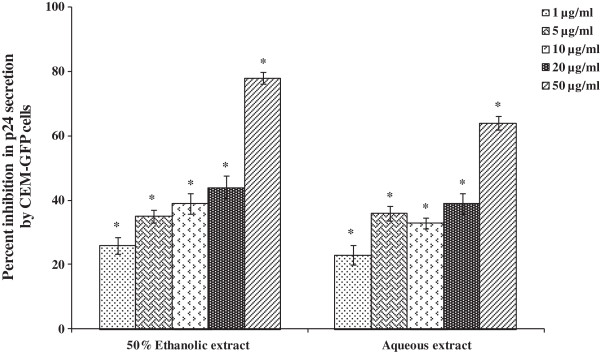
**Reduction in virus load by leaves extracts of *R. parviflora *using CEM-GFP cells.** Cells were infected with HIV-1_NL4.3_ (0.05 MOI) and treated with extracts at various concentrations followed by 8 day incubation. Culture supernatant was collected on 8^th^ day for p24 estimation by ELISA. AZT (10 μM) was used as positive control that reduced p24 level by 97%. Data is represented as mean ± SE of three independent experiments performed in duplicates. Statistical significance between the extract treated groups as compared to untreated control is shown by asterisk (*p < 0.001).

The inhibitory activity of the extracts against HIV infection is sometimes, a result of their toxic effects and consequently might result in an erroneous conclusion. Hence to exclude the non-specific antiviral effect, the toxicity of plant extracts on TZM-bl and CEM-GFP cells was assessed using MTT assay. The CC_50_ values of 50% ethanolic as well as aqueous extracts prepared from leaves of *R. parviflora* were > 331 μg/ml on TZM-bl cells and >196 μg/ml on CEM-GFP cells (Table 1). Both aqueous (IC_50_; 15 μg/ml) and 50% ethanolic extract (IC_50_; 26 μg/ml) showed dose dependent inhibition in HIV-1 infection using TZM-bl cell-based assay (Table 1). Treatment of HIV-1 infected CEM-GFP cells with the 50% ethanolic as well as aqueous extracts of the leaves of *R. parviflora* also showed a dose dependent inhibition of HIV infection with IC_50_ values of 29 and 65 μg/ml, respectively (Table 1). Both the extracts reduced the virus released by infected CEM-GFP cells as estimated by p24 in the culture supernatants collected from the infected cells (Figure 1). The higher efficacy of the extracts in TZM-bl cells as compared to CEM-GFP cells-based assay may be due to an additional step of virus pre-treatment with extracts in TZM-bl cells-based assay format and hence suggests the presence of additional virucidal phytochemicals. The therapeutic index (TI) of a drug is the ratio between the toxic and the therapeutic dose and is used as a measure of its relative safety. The TI values of the extracts were in a range of 6.8 to 25.5 (Table 1).

**Table 1:** ***In vitro *cytotoxicity and anti-HIV activity of the extracts derived from leaves of *R. parviflora *using TZM-bl and CEM-GFP cells**

**Plant extract**	**CC**_**50 **_(μg/ml)*	**IC**_**50 **_(μg/ml)*	**TI**
	**TZM-bl cells**		
**50% Ethanolic extract**	331	26	12.7
**Aqueous extract**	383	15	25.5
	**CEM-GFP cells**		
**50% Ethanolic extract**	196	29	6.8
**Aqueous extract**	643	65	9.9

*CC_50_: The cytotoxic concentration of the extracts that caused the reduction of viable cells by 50%. All data presented are averages of duplicate experiments.

*IC_50_: The concentration of the extracts that resulted in 50% inhibition in HIV infection. All data presented are averages of duplicate experiments.

TI: Therapeutic index is CC_50_/IC_50._

**Figure 2:**
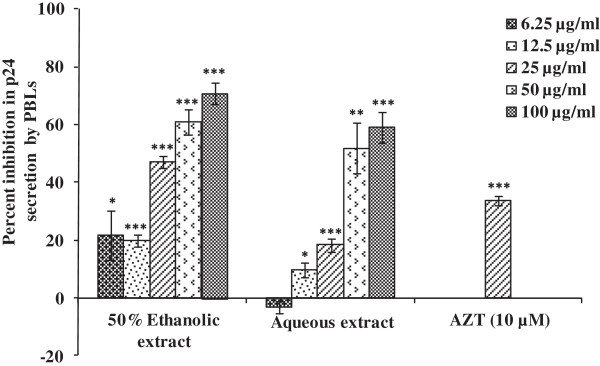
**Anti-HIV activity of leaves extracts of *R. parviflora *on infected human peripheral blood lymphocytes (PBLs).** Cells were infected with HIV-1_NL4.3_ (0.05 MOI) and treated with extracts at various concentrations followed by 5 days incubation. Culture supernatant was collected for p24 estimation by ELISA. AZT (10 μM) was used as positive control that reduced p24 level by 33%. Data is represented as mean ± SE of three independent experiments performed in duplicates. Statistical significance between the extract treated groups as compared to untreated control is shown by asterisk (*p < 0.05; **p < 0.01; ***p < 0.001).

Furthermore, apart from the usage of reporter-gene based assays, the inhibitory activity of the extracts from *R. parviflora* was also assessed using activated human lymphocytes (PBLs; biological targets of HIV including CD4^+^ T cells, monocytes, dendritic cells, etc.) from blood of HIV seronegative donors. The extracts were non-toxic to PBLs up to a concentration of 100 μg/ml (data not shown). There was a dose-dependent inhibition in p24 secretion by infected PBLs that were treated with 50% ethanolic and aqueous extracts prepared from leaves of *R. parviflora* (Figure 2). The present study demonstrates for the first time that leaves extracts prepared from *R. parviflora* display inhibitory effect on HIV-1 replication. Several phytochemicals such as gallic acid, myricetin, quercetin, kampferol and different glycosides have been reported to be present in *R. parviflora*[16,21]. Medicinally important compounds like gallic acid, quercetin, kampferol, glycosides etc. from other plants have been reported for their anti-HIV activity suggesting that the presence of these compounds may be responsible for the inhibition of HIV infection by *R. parviflora* leaves extracts [29,30]. However, further studies for identification and characterization of the anti-HIV active compounds from leaves extract of this plant are still required.

### Anti-HIV activity of the extracts from *R. parviflora* mediated by inhibiting HIV-1 protease activity

Since HIV-1 is a retrovirus, virally encoded enzyme reverse transcriptase (RT) that catalyzes the conversion of viral RNA to proviral DNA is an important target where the extract may act to inhibit HIV infection. For this, the RT activity was evaluated in presence and absence of extract using a kit (Roche Diagnostics). No inhibition in RT activity was observed in presence of both the extracts (50 μg/ml) as compared to Nevirapine (1 μM) used as a positive control with ~90% inhibition (data not shown). These results imply that anti-HIV activity of the leaves extract of *R. parviflora* is not mediated by inhibition of HIV-1 RT activity, rather the extract may act at different steps of HIV life cycle.

**Figure 3:**
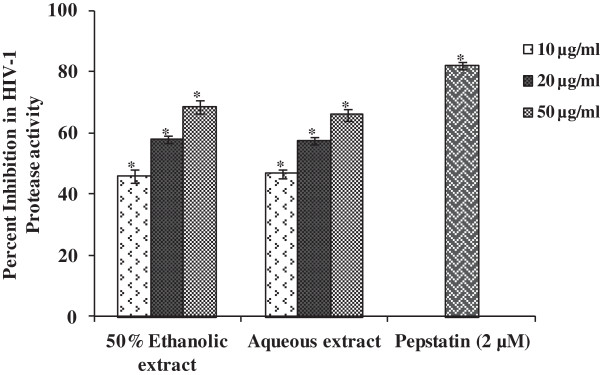
**Inhibitory effect of the extracts prepared from leaves of *R. parviflora *on HIV-1 protease activity.** Inhibition of HIV-1 protease by 50% ethanolic and aqueous extracts prepared from leaves of *R. parviflora* was determined by a commercial kit*.* Experiments were performed using both positive (HIV-1 protease with pepstatin treatment) as well as negative controls (HIV-1 protease without treatment). Data is expressed as percent inhibition of HIV-1 protease activity determined by dividing the difference in fluorescent intensity of experimental and negative control by negative control followed by multiplication with 100. Data is represented as mean ± SE of three independent experiments. Statistical significance between the extract treated groups as compared to untreated enzyme control group is presented by asterisk (*p < 0.001).

HIV-1 protease is another important enzyme of the HIV life cycle acting at post-entry level. Protease influences the viral components to associate with host cell membrane which then buds off as immature virions. Protease activity continues after detachment from the host cell to ensure maturation into a fully infectious virion. The 50% ethanolic and aqueous leaves extracts of *R. parviflora* were evaluated for their effect on HIV-1 protease activity. Both these extracts inhibited HIV-1 protease activity in a dose dependent manner when tested at 10, 20 and 50 μg/ml. A maximum inhibition of >65% was shown by both the extracts at 50 μg/ml (Figure 3). Hence, HIV-1 protease inhibition is one of the mode by which the extracts prepared from leaves of *R. parviflora* inhibited HIV infection. However, a higher degree of inhibition of HIV-1 infection by these extracts might be attributed to the presence of several phytochemicals that could work through interference at other steps of virus life cycle.

### Extracts derived from *R. parviflora* has no adverse effect on trans-epithelial cells resistance and viability of lactobacilli

**Figure 4:**
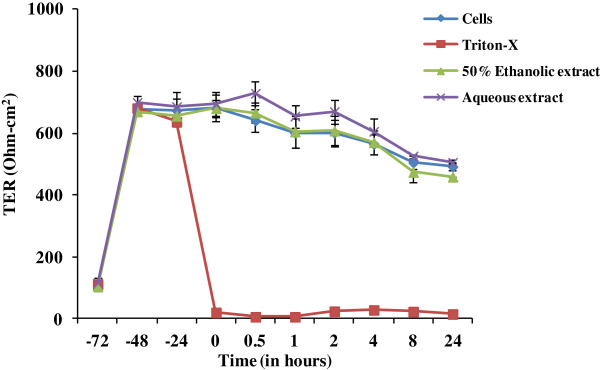
**Effect of the extracts of *R. parviflora *on epithelial monolayer integrity.** Caco-2 cells were grown in transwell supports until they formed stable monolayer. Plant extracts or vehicle control were added to the apical chamber at t = 0 and resistance was measured at 0.5, 1, 2, 4, 8 and 24 h. As toxicity reference control Triton-X (10%) was added to the indicated apical chambers whereas the test extracts were evaluated at 50 μg/ml. Data shown represent the mean ± SE of two independent experiments performed in duplicates.

Integrity of the epithelial cell lining of the reproductive tract is critical for the prevention of sexual transmission of HIV. The maintenance of an intact and polarized monolayer in presence of any topical agent used as microbicide indicates their safety on cervical and colorectal tissues [31]. The integrity of the intact epithelium as an effect of these plant extracts was evaluated by measuring transepithelial resistance (TER). Both 50% ethanolic and aqueous extracts were screened for their toxicity on Caco-2 cells, prior to TER measurement. TER was performed at a non-toxic concentration of the extracts (50 μg/ml). The extracts were added after the cells established a confluent monolayer, followed by TER measurement for 24 h. The non-toxic concentration of the extracts did not reduce the TER significantly (p > 0.05) as compared to the untreated controls. After 24 h of application, the TER value was same as of untreated cells except for Triton-X that led to a non-reversible reduction in TER even after half an hour of its application (Figure 4). Overall, the non-toxic concentration of the extracts of *R. parviflora* did not affect the TER and hence may be suitable candidates for topical application.

**Figure 5:**
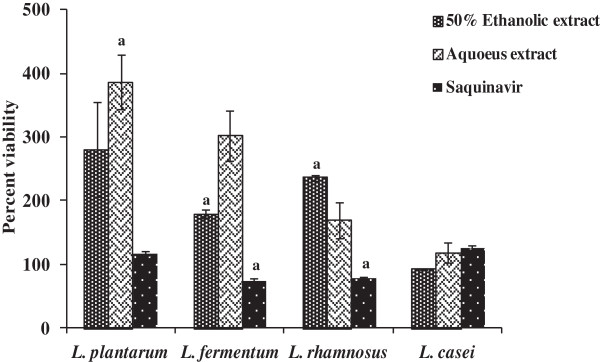
**Effect of leaves extracts derived from *R. parviflora *on different Lactobacillus strains.** Various lactobacilli strains (10^8^ CFU/ml) were cultured in presence or absence of various extracts/saquinavir (a commercially available HIV-1 protease inhibitor used as a reference control) at 100 μg/ml, for 24 h at 37°C and followed-up by viability assessment using MTT assay as described in *Methods*. Values are expressed as percent viability determined by dividing the absorbance of treated cells to untreated cells followed by multiplication with hundred. Data is represented as mean ± SE of three independent experiments performed in duplicates. Statistical significance of either the increase or decrease in viability of lactobacilli treated with either plant extracts or saquinavir as compared to untreated lactobacilli is shown by alphabets (^a^p < 0.05).

*Lactobacillu*s sp. are the dominant members of the human vaginal microflora, where they play a protective role against urogenital infection, as well as prevent attachment of HIV virus [32], so it is essential that the extracts from *R. parviflora* should be non-toxic to their growth. Incubation of *Lactobacillus casei*, *L. fermentum*, *L. plantarum* and *L. rhamnosus* with aqueous as well as 50% ethanolic extracts up to 100 μg/ml revealed no cytotoxic effects. Viability of different lactobacilli strains at 100 μg/ml of different extracts/saquinavir is shown in Figure 5. On comparison with saquinavir, a commercially used protease inhibitor, less than 75% viable population of *L. fermentum* and *L. rhamnosus* was recorded. Natural barriers of HIV are low pH, presence of lactobacilli, intact epithelial surface and the mucosal immune system of the genital tract [33,34]. Therefore, *in vitro* toxicity on lactobacilli may provide an effective and inexpensive method to evaluate the safety of candidate microbicides in a preclinical setting. *R. parviflora* have high levels of flavonoids and polyphenols [16] and according to previous studies, growth of lactobacilli are stimulated by catechins and polyphenol compounds [35,36].

### No significant increase in pro-inflammatory cytokines observed in human vaginal derived cells after treatment with extracts prepared from *R. parviflora*

While considering these extracts as candidates to be used for prevention of sexual transmission of HIV, their effect on primary vaginal keratinocytes viability is highly relevant, as vaginal epithelial cells form a part of the physical barrier that may impede the passage of cell-free or cell associated HIV-1 into sub epithelial tissues. Clinical trials based on N-9-containing microbicidal products have raised concerns that disruption of the cervicovaginal epithelium as well as rise in pro-inflammatory cytokines secretion by spermicides or microbicides application may increase the susceptibility to HIV-1 infection by providing a direct portal of entry for the virus to subcutaneous tissues and/or by recruiting HIV-1-susceptible immune cells to the genital tissues [37-39]. Therefore, ruling out an increase of pro-inflammatory cytokines secretion after application of the plant extracts on vaginal surface was particularly important. Vk2/E6E7 cells are immortalized human vaginal epithelial cells that proved to be an adequate model for studying the vaginal responses to topical agents [26]. No cytotoxicity was observed for Vk2/E6E7 cells, when these cells were cultured with the extracts up to 100 μg/ml for 24 h. The concentrations of IL-1β, IL-6, IL-8 and TNF were determined in the culture supernatants. As compared to the control, a decrease in the concentration of IL-1 β as well as IL-8 was observed subsequent to treatment of Vk2/E6E7 cells with both the extracts. However, treatment with 50% ethanolic extract led to a significant (p < 0.001) decrease in IL-8 secretion (Table 2). An increase in the secretion of IL-6 and TNF was observed which was non-significant and within the non-inflammatory range [40]. Thus, the extracts from *R. parviflora* appear to be safe and may be useful candidate to be developed as microbicides.

**Table 2:** **Pro-inflammatory cytokines secretion by vaginal keratinocytes (Vk2/E6E7) after treatment with extracts prepared from *R. parviflora *leaves**

**Treatment with plant extract**^**#**^	**Level of pro-inflammatory cytokines (pg/ml)**			
	**IL-1β**	**IL-6**	**IL-8**	**TNF**
**Control**	2.0 ± 0.2	119.0 ± 9.1	288.0 ± 5.3	1.1 ± 0.6
**50% Ethanolic extract**	1.7 ± 0.0	164.4 ± 10.0	10.4 ± 1.0*	1.7 ± 0.3
**Aqueous extract**	1.6 ± 0.6	126.7 ± 9.4	241.5 ± 10.2	1.5 ± 0.4

^**#**^100 μg/ml for 24 h.

*p < 0.001 as compared to IL-8 concentration in the control group.

## Conclusions

In addition to its other traditional uses, the extracts prepared from *R. parviflora* have anti-HIV-1 property, which may be mediated through inhibition of HIV-1 protease activity. The extracts have no adverse effect on the growth of lactobacilli, epithelial monolayer integrity and subsequent to treatment with the extracts, pro-inflammatory cytokines levels are within the non-inflammatory range. These observations are encouraging and further safety and efficacy studies *in vivo* may be undertaken to explore potential of the extracts prepared from *R. parviflora* for prevention of HIV sexual transmission.

## Competing interests

The authors declare that they have no competing interests.

## Authors’ contributions

SKG designed and coordinated the overall study and wrote the manuscript. SM coordinated with AKS and SK for collection and extraction of plant material. SKG, MM, N and BP designed and performed the HIV-1 and safety experiments, as well as analysed the data. All authors read and approved the final manuscript.

## Pre-publication history

The pre-publication history for this paper can be accessed here:

http://www.biomedcentral.com/1472-6882/13/158/prepub

## Supplementary Material

Additional file 1: Figure S1 HPLC profiles of aqueous and 50% ethanolic extracts prepared from leaves of *R. parviflora*. HPLC was performed using C18 column (4.6 mm × 250 mm) at a flow rate of 0.4 ml/min. An isocratic elution (acetonitrile-water with 10 mM of formic acid; 35:65) was performed and peaks were monitored at 280 nm. Figure A represents HPLC profile of aqueous extract (20 μg) and Figure B represents profile of 50% ethanolic extract (20 μg). Click here for file
