# Supplementary figures and images for: Anti-HIV-1 activity, protease inhibition and safety profile of extracts prepared from Rhus parviflora
Source: BMC Complement Altern Med. 2013 Jul 4;13:158. doi: 10.1186/1472-6882-13-158 (PMC3716979; doi:10.1186/1472-6882-13-158)

## Slide 1
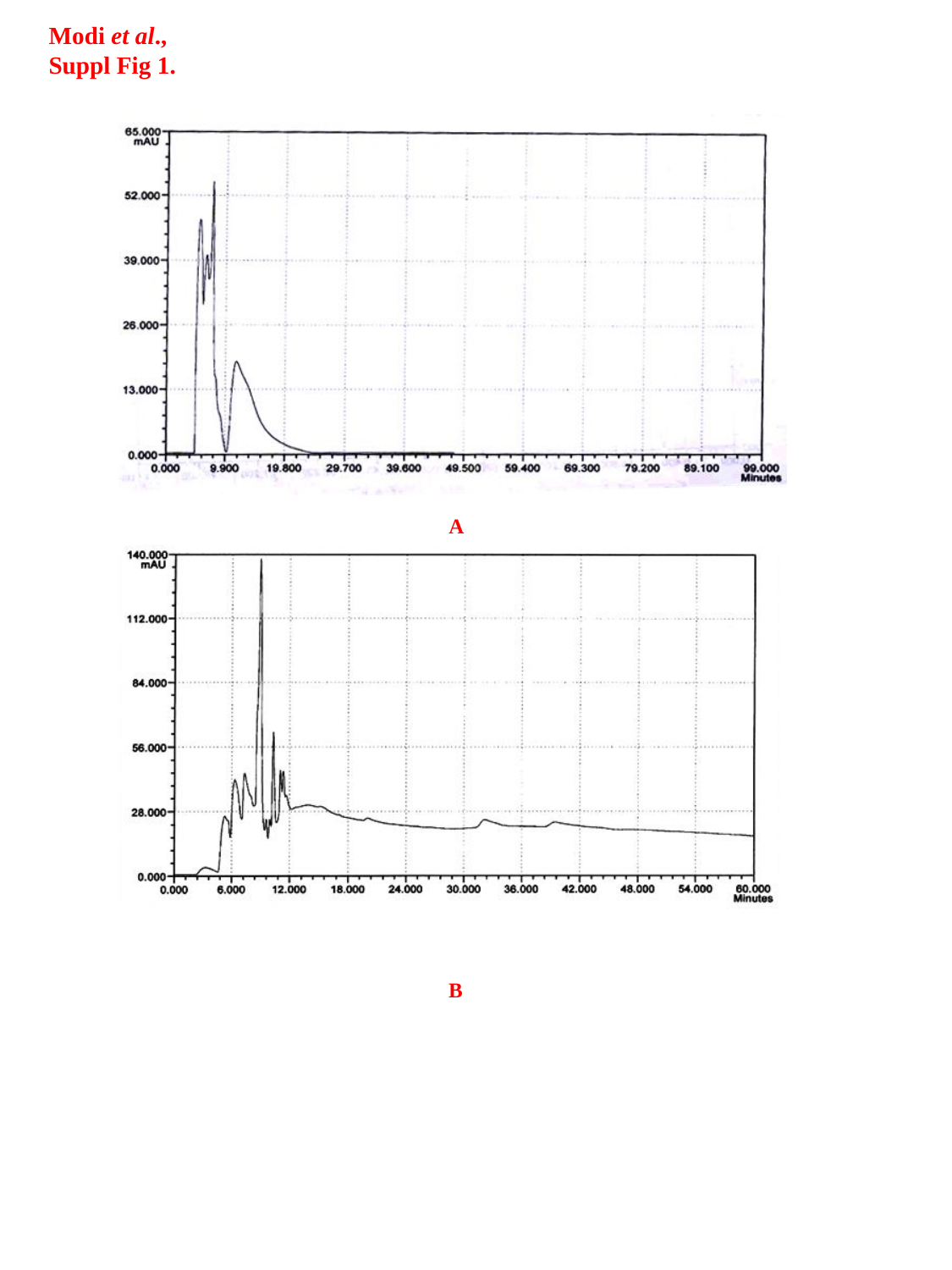

Modi et al.,
Suppl Fig 1.
A
B

Supplement: Additional file 1: Figure S1 — HPLC profiles of aqueous and 50% ethanolic extracts prepared from leaves of R. parviflora. HPLC was performed using C18 column (4.6 mm × 250 mm) at a flow rate of 0.4 ml/min. An isocratic elution (acetonitrile-water with 10 mM of formic acid; 35:65) was performed and peaks were monitored at 280 nm. Figure A represents HPLC profile of aqueous extract (20 μg) and Figure B represents profile of 50% ethanolic extract (20 μg). [file 1472-6882-13-158-S1.pptx]
